# Diffusion tensor image analysis along the perivascular space (DTI-ALPS) in Parkinson’s disease patients with subjective cognitive decline

**DOI:** 10.3389/fnagi.2026.1935631

**Published:** 2026-09-18

**Authors:** Shanzhen Wei, Mengdi Qiu, Mengyu Gong, Wenwei Zou, Jiaxin Ren, Wenchao Qiu

**Affiliations:** 1 Department of Electrophysiology, The Affiliated Huai’an Hospital of Xuzhou Medical University, Huai’an, China; 2 Department of Neurology, The Fifth People’s Hospital of Huai’an, Huai’an, China; 3 Department of Neurology, The Affiliated Huai’an Hospital of Xuzhou Medical University, Huai’an, China; 4 Department of Neurology, Lianshui County People’s Hospital, Huai’an, China

**Keywords:** attention and working memory, DTI-ALPS, glymphatic system, Parkinson’s disease, subjective cognitive decline

## Abstract

**Introduction:**

This study aimed to examine diffusion alterations indexed by the diffusion tensor image analysis along the perivascular space (DTI-ALPS) in patients with Parkinson’s disease presenting with subjective cognitive decline (PD-SCD), and to investigate their associations with impairments across specific cognitive domains.

**Methods:**

This is a cross-sectional study. A total of 607 eligible participants were enrolled and categorized into three groups: healthy controls (HC), Parkinson’s disease with normal cognition (PD-NC), and PD-SCD. The DTI-ALPS index was calculated as a diffusion-based metric reflecting water diffusivity along the perivascular space relative to adjacent fiber tracts. To ensure the robustness of the findings, Region of Interest (ROI) delineation was performed and validated using both manual anatomical delineation and JHU atlas-based methods. Statistical analyses included inter-group comparisons, Receiver Operating Characteristic (ROC) curves to assess predictive performance for PD-SCD, and partial correlation analyses between ALPS indices and cognitive domains.

**Results:**

The DTI-ALPS indices were significantly lower in the PD-SCD group compared to both PD-NC and HC groups, exhibiting a significant stepwise decline (HC > PD-NC > PD-SCD). ROC analysis showed that the left ALPS index (AUC = 0.6503) and the mean ALPS index (AUC = 0.6452) had modest discriminatory performance in differentiating PD-SCD from PD-NC. Partial correlation analysis revealed that ALPS indices were specifically and significantly associated with attention and working memory deficits. Notably, the side of motor symptom onset in PD did not significantly influence ALPS values. High consistency was observed between the manual and atlas-based ROI methods.

**Discussion:**

In this cross-sectional cohort, PD-SCD was associated with a lower DTI-ALPS index, and this diffusion alteration was related to attention and working-memory performance. These findings suggest that the DTI-ALPS index may serve as a potential diffusion-based marker for early cognitive risk stratification in PD; however, its precise biological basis and longitudinal significance require further investigation.

## 1 Introduction

Parkinson’s disease (PD) is the second most prevalent neurodegenerative disorder worldwide, with motor manifestations including bradykinesia, resting tremor, rigidity, and postural instability (Kalia and Lang, 2015). However, non-motor symptoms also exert an impact on patients’ quality of life that often equals or exceeds that of motor dysfunction (Chaudhuri and Schapira, 2009). Among these non-motor symptoms, cognitive impairment is a particularly debilitating feature, with prevalence escalating alongside disease progression, thereby serving as a critical predictor of disability and mortality (Aarsland et al., 2017). The cognitive spectrum in PD is heterogeneous, spanning from mild cognitive impairment (PD-MCI) to Parkinson’s disease dementia (PDD) (Jamora et al., 2025; Kalia and Lang, 2015).

Subjective cognitive decline (SCD) represents the earliest stage within this progressive cognitive continuum. Clinically, SCD is characterized by a patient’s self-reported, persistent decline in cognitive capacity, despite objective neuropsychological performance falling within age- and education-adjusted norms (Stewart, 2012). In this study, we employed the Cognition Complaint Interview (CCI) to assess SCD (Dujardin et al., 2010; Thomas-Antérion et al., 2006). Unlike broader screening tools such as the Cognitive Decline Questionnaire (CDQ) or visual analog scales (Dupouy et al., 2018; Ophey et al., 2022; Siciliano et al., 2020), the CCI provides a multi-dimensional framework that systematically characterizes the nature, duration, and functional consequences of cognitive complaints, thereby ensuring greater diagnostic specificity and cohort homogeneity.

Subjective cognitive decline was initially characterized as a preclinical prodromal marker for Alzheimer’s disease (AD) (Jessen et al., 2014). It is not merely a psychological symptom of aging or anxiety, but rather a tangible early indicator of underlying neurodegeneration (An et al., 2024). Longitudinal data indicate that PD patients reporting SCD are at a significantly heightened risk of progression to objective MCI or dementia (Galtier et al., 2019; Huang et al., 2023). Given that PD-SCD may harbor early neuroimaging or biochemical signatures of decline, it offers a vital “window of opportunity” for early identification and therapeutic intervention (Rodriguez-Antiguedad et al., 2025; Stav et al., 2015; Yoo et al., 2021). However, the neurobiological mechanisms defining this stage–specifically the link between imaging markers and cognitive deterioration–remain incompletely understood.

Traditional research into PD cognitive decline has largely focused on α-synucleinopathy, neurotransmitter deficits, neuroinflammation, and oxidative stress. Recently, the characterization of the glymphatic system–a specialized waste clearance network within the brain–has provided a transformative perspective on neurodegenerative pathology (Jessen et al., 2015). First described in rodents by Iliff et al. (2012), this system facilitates the exchange between cerebrospinal fluid (CSF) and interstitial fluid (ISF), thereby promoting the clearance of metabolic waste and toxic protein aggregates. This process is highly dependent on sleep architecture, peaking during slow-wave sleep when interstitial spaces expand to facilitate efficient fluid flux (Xie et al., 2013).

Glymphatic dysfunction is increasingly implicated in neurodegeneration, where impaired clearance promotes toxic protein accumulation, subsequent neuroinflammation, and accelerated neuronal injury (Zamani et al., 2022; Zhang Q. et al., 2025). Intrathecal tracer-based MRI or CT cisternography remains the gold standard for direct visualization of CSF-ISF exchange, but its inherent invasiveness precludes routine clinical application or large-scale screening (Albayram et al., 2008; Algin and Turkbey, 2013; Yoo et al., 2008). The diffusion tensor image analysis along the perivascular space (DTI-ALPS) index was developed as a non-invasive, diffusion-based surrogate marker (Taoka et al., 2017). This technique leverages the principles of Diffusion Tensor Imaging (DTI), which measures the anisotropy of water molecule diffusion (Basser et al., 1994; Le Bihan et al., 2001). Medullary perivascular spaces (PVS) run parallel to deep medullary veins and sit perpendicular to ventricular white matter projection/association bundles, and unrestricted water diffusion can be reliably captured on DTI sequences. The ALPS index is calculated by comparing diffusivity along the PVS (*x*-axis) to that perpendicular to both projection and association fibers (Taoka et al., 2017). This technique has demonstrated diagnostic promise in AD, PD, and sleep disorders (Bae et al., 2023; Marecek et al., 2025; Zhang Q. et al., 2025). However, systematic research focusing specifically on the PD-SCD subpopulation remains sparse, particularly regarding the characteristic changes in DTI-ALPS and their correlations with specific cognitive domain deficits. It is important to note that DTI-ALPS is an indirect, ratio-based diffusion metric obtained at a specific anatomical location. Its interpretation has recently been revisited: although a reduced ALPS index has frequently been regarded as reflecting impaired glymphatic clearance, it may also be influenced by the organization and integrity of projection and association fibers, extracellular water content, regional tissue geometry, and other microstructural properties (Taoka et al., 2024, 2026). Moreover, a recent study comparing DTI-ALPS with intrathecal contrast-enhanced MRI measures of CSF tracer enrichment found only limited correspondence between the two approaches, suggesting that the ALPS index may not directly reflect glymphatic function as ascertained by tracer-based methods (Mossige et al., 2026). Accordingly, in the present study we describe our findings primarily in terms of higher or lower ALPS indices and discuss a possible relationship with the glymphatic system only as an interpretation, with the relevant methodological limitations explicitly acknowledged.

This study was designed to bridge these gaps by evaluating the clinical utility of the DTI-ALPS index in patients with PD-SCD. We aim to provide robust evidence for DTI-ALPS as an early imaging biomarker for PD-SCD.

## 2 Materials and methods

### 2.1 Participants

We conducted a cross-sectional imaging cohort study to investigate the clinical utility of the DTI-ALPS index in patients with PD-SCD. Participants were categorized into three cohorts: healthy controls (HC), PD patients with normal cognition (PD-NC), and PD patients with subjective cognitive decline (PD-SCD) (Figure 1). This study was approved by the Ethics Committee of the Affiliated Huai’an Hospital of Xuzhou Medical University and was conducted in strict accordance with the Declaration of Helsinki. Written informed consent was obtained from all participants prior to their enrollment.

**FIGURE 1:**
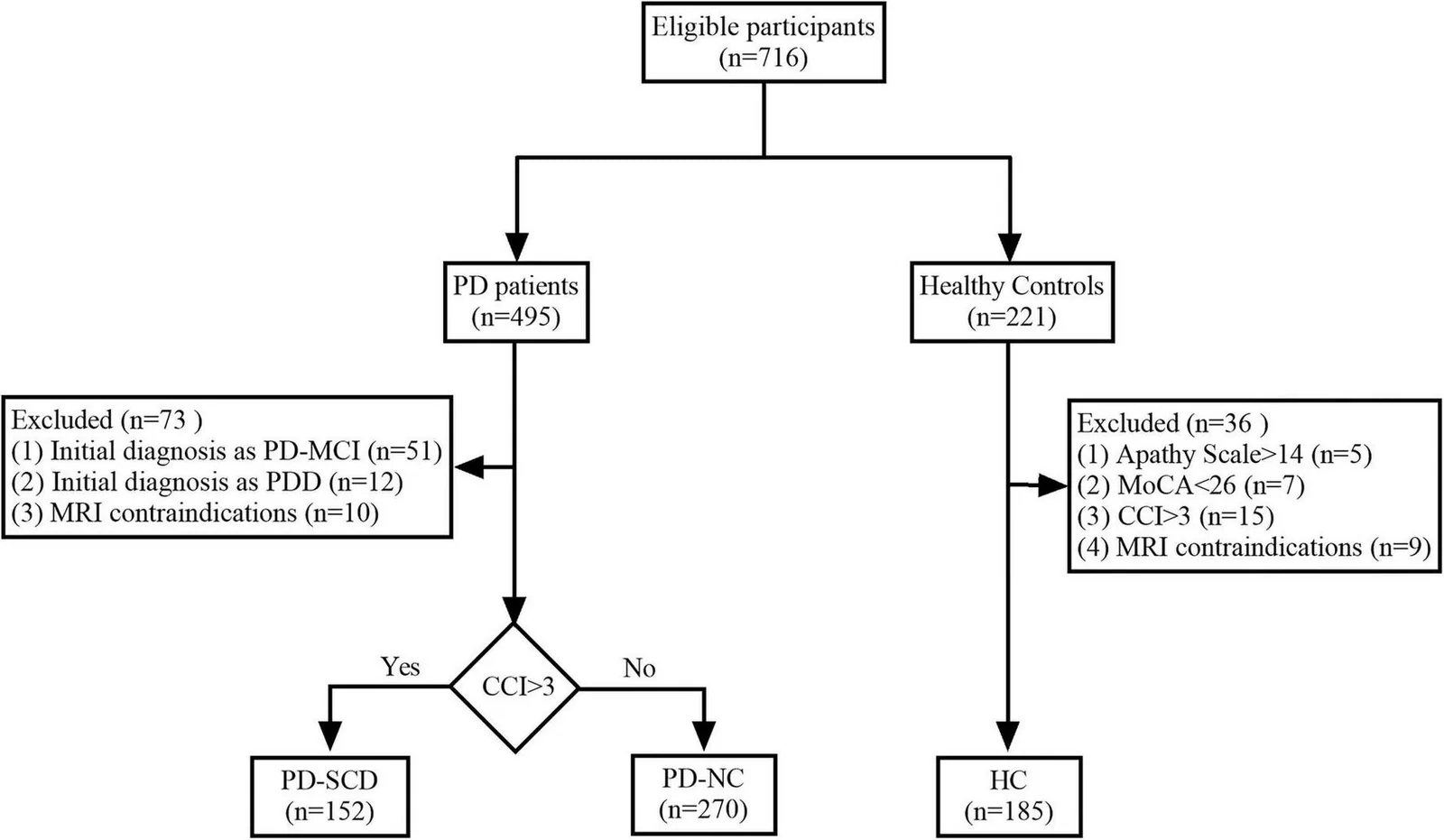
Flowchart of participants enrollment and classification. PD, Parkinson’s disease; PD-SCD, Parkinson’s disease with subjective cognitive decline; PD-NC, Parkinson’s disease with normal cognition; HC, healthy controls; PD-MCI, Parkinson’s disease with mild cognitive impairment; PDD, Parkinson’s disease dementia; MoCA, Montreal Cognitive Assessment; CCI, Cognition Complaint Interview.

Parkinson’s disease patients were recruited from the Department of Neurology, while healthy controls were recruited from our institution’s Health Examination Center. All PD patients underwent MRI and neuropsychological assessment in the on-medication state. Comprehensive clinical and neuropsychological evaluations were performed by experienced neurologists and neuropsychologists.

Criteria for inclusion in the HC group included age between 50 and 80 years, no history of significant neurological or psychiatric conditions, and objective cognitive performance within normal limits, defined as a Montreal Cognitive Assessment (MoCA) score ≥ 26 and a Cognition Complaint Interview (CCI) score ≤ 3 (Thomas-Antérion et al., 2006). Patients in the PD-NC group were required to meet the 2015 Movement Disorder Society (MDS) clinical diagnostic criteria for PD, alongside normal cognitive scores (MoCA ≥ 26; CCI ≤ 3) (Postuma et al., 2015). Patients in the PD-SCD group met the same MDS diagnostic criteria for PD and age requirements, but with a CCI score > 3, reflecting self-reported cognitive decline for at least 6 months that clinicians deemed inconsistent with normal aging.

Exclusion criteria were applied universally across all groups. Participants were ineligible if they presented with MRI contraindications, a history of major neurological disorders, or severe psychiatric illness. Additionally, individuals with current severe depression or anxiety (HAMD-24 score ≥ 20; HAMA-14 score ≥ 15), significant apathy (Apathy Scale > 14), history of substance abuse, severe systemic diseases, or the use of medications known to influence cognitive performance were excluded.

### 2.2 Neuropsychological assessment

All participants completed a standardized neuropsychological test battery administered by trained assessors. This battery assessed five distinct cognitive domains: attention and working memory, executive function, memory, language, and visuospatial ability. Attention and working memory were evaluated via the Digit Span Test (backward) and the Digit Symbol Test (Beck et al., 1962; Heilbrun, 1958). Executive function was assessed via the Trail Making Test Part B (TMT-B) and the Stroop Color-Word Test (Lu and Bigler, 2000; Van der Elst et al., 2006). Memory performance was measured by the Rey Auditory Verbal Learning Test (RAVLT) delayed recall and the Huashan version of the Auditory Verbal Learning Test (AVLT-H) recognition subtest (Qinjie et al., 2016; Rosenberg et al., 1984). Language ability was assessed using the Boston Naming Test (BNT-30) and the Verbal Fluency Test (animal category) (Li et al., 2022; Sciarma et al., 1990; Villalobos et al., 2023), while the Clock Copying Test (CLOX2) and Benton Judgment of Line Orientation Test (BJLOT-30) were employed to quantify visuospatial ability (Benton et al., 1978; Royall et al., 1998).

To account for heterogeneity across different test scales and raw scores, all data were transformed into Z-scores based on the mean and standard deviation of the HC group. For each cognitive domain, a composite Z-score was derived by averaging the Z-scores of the constituent tests, providing a standardized basis for subsequent statistical analysis.

### 2.3 Neuroimaging data acquisition

All magnetic resonance imaging (MRI) data were acquired using a 3.0T Siemens Prisma scanner. A standardized imaging protocol was strictly followed to ensure consistency across all participants. T1-weighted imaging (T1WI) parameters were as follows: repetition time (TR) = 2300 ms, echo time (TE) = 2.98 ms, flip angle = 8°, field of view (FOV) = 256 mm × 256 mm, matrix = 256 × 256, and slice thickness = 1 mm.

Diffusion tensor imaging (DTI) data were acquired using a single-shot echo-planar imaging sequence with 45 axial slices, 3 mm slice thickness, and no gap. The acquisition parameters were: TR = 6600 ms, TE = 93 ms, and an acquisition matrix of 128 × 128. Diffusion gradients were applied in 30 directions with a *b*-value of 1000 s/mm^2^, accompanied by one *b* = 0 s/mm^2^ volume. To minimize head motion artifacts, all participants were immobilized with foam pads during the scanning procedure. To mitigate motion-related artifacts, all volumes were visually inspected for excessive motion during preprocessing, and participants with evident motion artifacts or poor tensor fitting were excluded from further analysis.

### 2.4 DTI data preprocessing and DTI-ALPS calculation

The acquired DTI data were processed using DTI Studio^1^. Following motion and eddy current correction, diffusion tensor images were reconstructed, and fractional anisotropy (FA) maps and tensor component maps (including Dxx, Dyy, Dzz) were generated.

We utilized two complementary strategies for Region of Interest (ROI) definition to ensure the robustness of our findings. The primary approach was manual ROI placement: an experienced neuroradiologist (with 10 years of clinical experience), blinded to all clinical and demographic information, manually defined four symmetrical circular ROIs (5 mm diameter) on the color-coded FA map at the level of the lateral ventricular body, targeting the projection and association fiber regions in both hemispheres (Figure 2). To validate these results, we employed an atlas-based approach using the JHU-ICBM-81 DTI Atlas. Individual FA maps were first linearly registered to the JHU-ICBM-FA template, followed by nonlinear registration to account for residual anatomical differences. Registration quality was visually inspected for each participant, and registrations with obvious misalignment were repeated or the corresponding participant was excluded. ROIs from the template space were transformed into each individual’s native diffusion space using the inverse transformation, and the DTI-ALPS index was then calculated in native space using the same fiber-orientation definitions as in the manual approach. Partial-volume effects were minimized by restricting ROI placement to the center of the superior corona radiata and the superior longitudinal fasciculus, away from tissue boundaries. Any registration failure or ROI placement falling outside the brain parenchyma was treated as a quality-control failure and the corresponding measurement was excluded.

**FIGURE 2:**
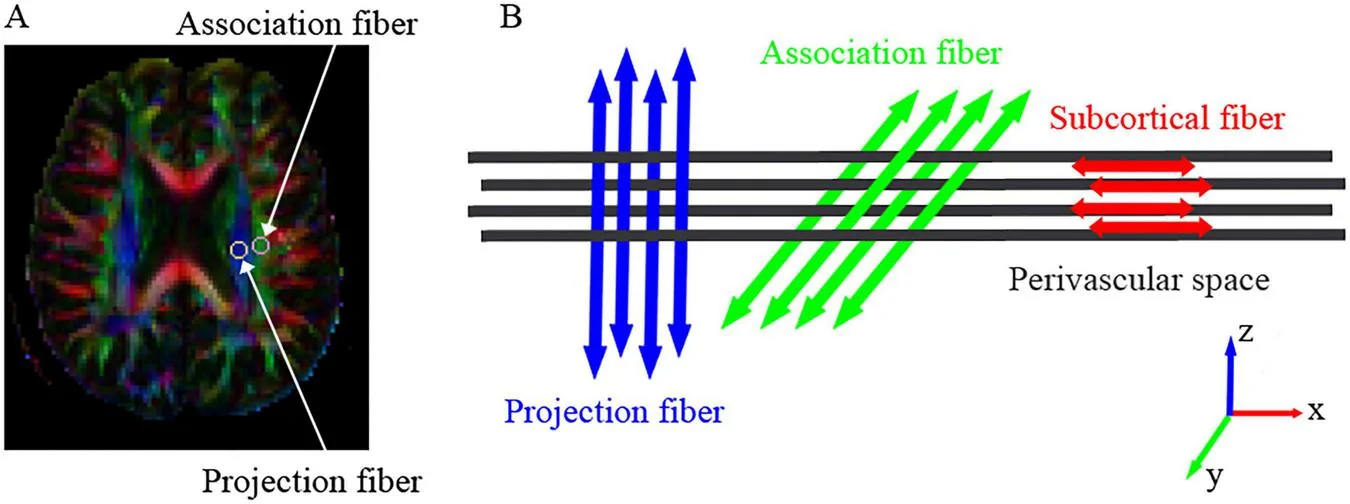
Visualization of three categories of neural fiber tracts at the level of the lateral ventricular body. **(A)** Color-coded FA maps display fiber orientation: projection fibers (blue, *Z*-axis), association fibers (green, *Y*-axis), and subcortical fibers (red, *X*-axis). ROIs (5 mm in diameter) were delineated within projection and association fiber bundles to quantify diffusion coefficients. **(B)** Diagram demonstrating the spatial relationship between perivascular spaces (gray columns) and neural fibers. The orientation of perivascular spaces is perpendicular to that of projection and association tracts.

The DTI-ALPS index was calculated according to the following formula (Taoka et al., 2017):


$$\mathrm{DTI}-\mathrm{ALPS}=\frac{\mathrm{mean}\,\left(D_{x\,x\,\_\,\mathrm{proj}},D_{x\,x\,\_\,\mathrm{assoc}}\right)}{\mathrm{mean}\,\left(D_{y\,y\,\_\,\mathrm{proj}},D_{z\,z\,\_\,\mathrm{assoc}}\right)}$$ (1)

This formula quantifies the diffusivity along the perivascular space (PVS) relative to the transverse diffusivity of the fiber bundles, thereby serving as an indirect measure of glymphatic system function. We calculated the index for both the left hemispheres (ALPS_L) and right hemispheres (ALPS_R), as well as the mean global ALPS index (ALPS_m).

### 2.5 Statistical analysis

Statistical analyses were performed using SPSS 26.0 and GraphPad Prism 9.5, and verified with Python 3.12. All statistical tests adopted two-sided testing, with *p* < 0.05 defined as the threshold for statistical significance. Group comparisons of the ALPS indices (ALPS_L, ALPS_R, and ALPS_m) among the three cohorts were conducted using one-way analysis of variance (ANOVA) followed by Bonferroni *post-hoc* tests. To address potential confounding by demographic and clinical differences between groups, we additionally performed analyses of covariance (ANCOVA). Because disease duration, motor severity (MDS-UPDRS III), and LEDD were not applicable to healthy controls, a three-group ANCOVA adjusting for age, sex, education, HAMA-14, HAMD-24, and Apathy-14 was performed across all cohorts, and a complementary ANCOVA restricted to PD patients additionally adjusted for disease duration, MDS-UPDRS III score, and LEDD. To investigate the potential effect of motor symptom onset side on glymphatic function, we compared ALPS indices between PD patients with left-sided (PD_L) and right-sided (PD_R) onset using independent samples *t*-tests. In addition, to formally test whether the left–right difference in ALPS indices differed between PD-NC and PD-SCD, we fitted a linear mixed-effects model with a random intercept per participant and fixed effects of group, hemisphere (left vs. right), and their interaction. The diagnostic performance of the ALPS index in distinguishing between PD-NC and PD-SCD patients was evaluated using Receiver Operating Characteristic (ROC) curve analysis, calculating the Area Under the Curve (AUC), sensitivity, and specificity. Finally, partial correlation analysis was performed to explore the relationship between ALPS indices and cognitive domain Z-scores, adjusting for age, sex, education, disease duration, HAMA-14, HAMD-24, and Apathy-14 scores to account for potential confounding effects. Given the exploratory nature of these analyses and the number of comparisons performed, false discovery rate (FDR) correction using the Benjamini–Hochberg procedure was applied to the resulting *p*-values.

## 3 Results

### 3.1 Demographic, clinical, and cognitive characteristics

A total of 607 participants were included in this study, comprising 152 in the PD-SCD group, 270 in the PD-NC group, and 185 in the HC group (Table 1). Baseline age and total PD disease duration were comparable across all cohorts (age *p* = 0.1411; disease duration *p* = 0.8267). However, the PD-SCD group exhibited a significantly higher proportion of males (70.4%) compared to the PD-NC (49.6%) and HC (53.0%) groups (*p* < 0.0001). Furthermore, the PD-SCD group had a lower level of education (5.92 ± 2.75 years) compared to both the PD-NC (7.95 ± 2.98 years) and HC (7.14 ± 2.89 years) cohorts (*p* < 0.0001).

**TABLE 1:** Demographic characteristics of the PD patients and healthy controls.

Demographic characteristics	PD-SCD *n* = 152	PD-NC *n* = 270	HC *n* = 185	*P*-value	*P*-value		
					PD-NC vs. PD-SCD	PD-NC vs. HC	PD-SCD vs. HC
Age, y	64.66 ± 6.46	63.11 ± 7.62	63.54 ± 6.27	0.1411	–	–	–
Age at onset, y	63.08 ± 6.73	60.68 ± 6.68	–	<0.0001	–	–	–
Male	107 (70.4%)	134 (49.6%)	98 (53.0%)	<0.0001	<0.0001	0.4835	0.0011
Education, y	5.92 ± 2.75	7.95 ± 2.98	7.14 ± 2.89	<0.0001	<0.0001	0.0109	<0.0001
Disease duration, m	25.09 ± 12.39	26.14 ± 12.42	–	0.8267	–	–	–
LEDD, mg	196.09 ± 104.38	166.24 ± 95.80	–	0.0001	–	–	–
Hypertension	43 (28.3%)	57 (21.1%)	49 (26.5%)	0.1973	–	–	–
Diabetes mellitus	6 (3.9%)	6 (2.2%)	11 (5.9%)	0.1231	–	–	–
Hyperlipidemia	17 (11.2%)	45 (16.7%)	32 (17.3%)	0.2345	–	–	–
Smoking	29 (19.1%)	46 (17.0%)	45 (24.3%)	0.1544	–	–	–
Drinking	21 (13.8%)	35 (13.0%)	27 (14.6%)	0.8820	–	–	–
MDS-UPDRS III score	18.07 ± 2.29	16.07 ± 2.05	–	<0.0001	–	–	–
MoCA score	28.03 ± 0.59	27.68 ± 0.58	28.08 ± 0.66	<0.0001	<0.0001	<0.0001	0.4141
HAMA-14	5.36 ± 1.85	5.49 ± 1.80	3.44 ± 0.91	<0.0001	0.9999	<0.0001	<0.0001
HAMD-24	8.03 ± 2.36	7.68 ± 2.46	3.97 ± 1.41	<0.0001	0.9999	<0.0001	<0.0001
Apathy-14	10.41 ± 3.24	10.31 ± 3.01	9.06 ± 2.28	<0.0001	0.9999	<0.0001	<0.0001
Executive function z score	0.2013 ± 0.6450	0.0826 ± 0.6570	0 ± 0.6909	0.0221	0.2361	0.5805	0.0175
Attention/working memory z score	−0.1260 ± 0.7052	−0.0524 ± 0.6886	0 ± 0.7135	0.2591	–	–	–
Language z score	−0.2553 ± 1.1760	−0.1207 ± 1.3658	0 ± 1.1358	0.1774	–	–	–
Memory z score	−0.2302 ± 1.2675	−0.0015 ± 1.0339	0 ± 1.1037	0.0917	–	–	–
Visuospatial z score	−0.2393 ± 0.7997	−0.1584 ± 0.9157	0 ± 1.0661	0.0540	–	–	–
Composite cognitive z score	−0.1299 ± 0.4292	−0.0501 ± 0.4377	0 ± 0.4261	0.0229	0.2070	0.6745	0.0186

SCD, Subjective Cognitive Decline; y, years; m, months; LEDD, Levodopa Equivalent Daily Dose; MDS-UPDRS, Movement Disorder Society Unified Parkinson’s Disease Rating Scale; MoCA, Montreal Cognitive Assessment.

When evaluating PD-specific clinical markers, the age of onset in the PD-SCD group (63.08 ± 6.73 years) was significantly higher than that in the PD-NC group (60.68 ± 6.68 years, *p* < 0.0001). Both LEDD (*p* = 0.0001) and MDS-UPDRS III scores (*p* < 0.0001) were significantly higher in the PD-SCD group compared to the PD-NC group. No intergroup disparities in vascular comorbidities (hypertension, diabetes, hyperlipidemia) or modifiable lifestyle exposures (smoking, alcohol consumption) were detected (*p* > 0.05).

The MoCA scores differed significantly across groups (*p* < 0.0001). *Post hoc* analyses revealed that only the PD-NC group (27.68 ± 0.58) scored significantly lower than both the HC (28.08 ± 0.66, *p* < 0.0001) and PD-SCD (28.03 ± 0.59, *p* < 0.0001) groups. No significant difference was detected between the HC and PD-SCD groups (*p* = 0.4141). As to neuropsychiatric symptoms, patients in both PD groups scored higher on the HAMA-14, HAMD-24, and Apathy-14 scales compared to the HC group (*p* < 0.0001). No significant differences were found between the PD-NC and PD-SCD subgroups (*p* > 0.9999).

Analysis of domain-specific Z-scores revealed that the PD-SCD group exhibited a lower composite Z-score (−0.1299 ± 0.4292) compared to the PD-NC (−0.0501 ± 0.4377) and HC (0 ± 0.4261) groups (*p* = 0.0229). Specifically, the PD-SCD group showed significantly impaired executive function (0.2013 ± 0.6450) relative to the HC group (0 ± 0.6909, *p* = 0.0175). No significant inter-group differences were observed in other cognitive domains, including attention/working memory, language, memory, and visuospatial ability.

### 3.2 Between-group comparisons of DTI-ALPS indices

Statistically significant differences in all DTI-ALPS indices among the three groups were observed in Figure 3 (all *p* < 0.0001). The ALPS_L index demonstrated a stepwise decline (HC: 1.353 ± 0.1081 > PD-NC: 1.321 ± 0.1266 > PD-SCD: 1.251 ± 0.1216). Specifically, the ALPS_L index was significantly lower in the PD-SCD group than in the PD-NC (*p* < 0.0001) and HC (*p* < 0.0001) groups. Similarly, the ALPS_m index showed a consistent pattern, with the PD-SCD group (1.258 ± 0.0902) exhibiting lower values than the PD-NC (1.305 ± 0.0847) and HC (1.346 ± 0.0858) groups (*p* < 0.0001). While the ALPS_R index was also significantly lower in the PD-SCD group compared to the HC group, no significant difference was observed between the PD-NC and PD-SCD subgroups (*p* = 0.1703). After adjustment for age, sex, education, and neuropsychiatric scores (HAMA-14, HAMD-24, Apathy-14), the three-group comparison confirmed significant overall group differences for ALPS_L, ALPS_R, and ALPS_m (all *p* < 0.001), with PD-SCD showing significantly lower ALPS_L (adjusted mean difference vs. PD-NC: −0.070; *p* < 0.001) and ALPS_m (adjusted difference: −0.046; *p* < 0.001) than PD-NC. In the PD-restricted model additionally adjusting for disease duration, MDS-UPDRS III score, and LEDD, the difference between PD-SCD and PD-NC remained significant for ALPS_L (β = −0.082, 95% CI −0.112 to −0.053, *p* < 0.001) and ALPS_m (β = −0.053, 95% CI −0.073 to −0.032, *p* < 0.001), but not for ALPS_R (β = −0.024, 95% CI −0.053 to 0.006, *p* = 0.120) (Supplementary Table 3).

**FIGURE 3:**
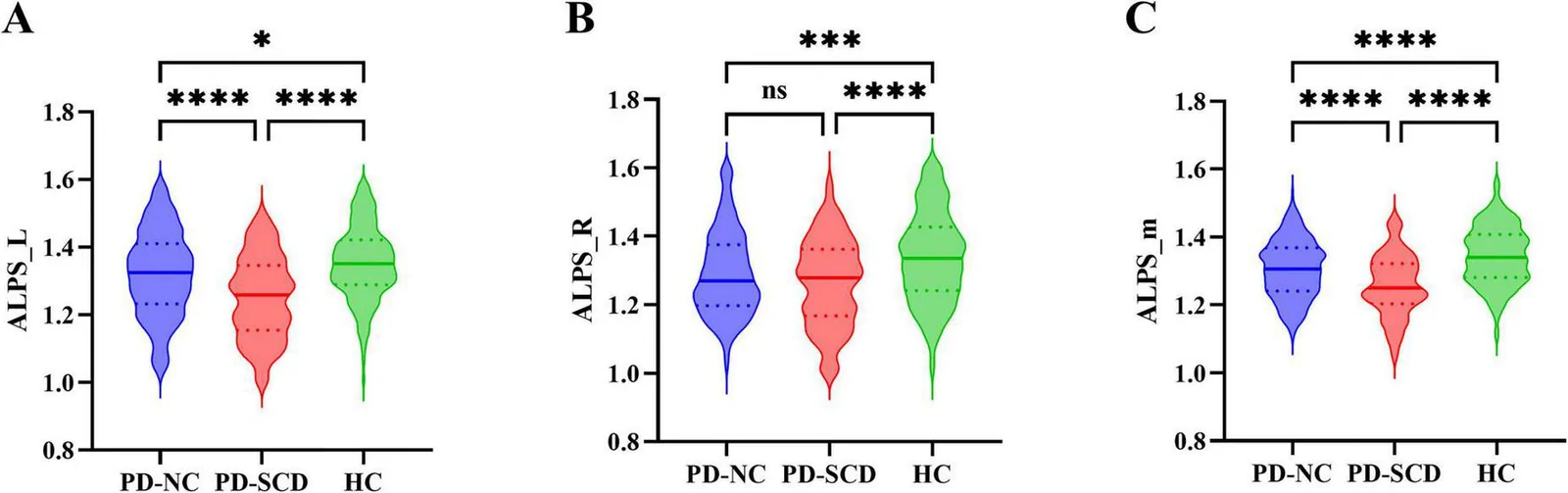
Violin plots showing group-wise distribution of DTI-ALPS indices. **(A)** ALPS_L decreased sequentially HC > PD-NC > PD-SCD. **(B)** PD-SCD showed lower ALPS_R than HC, with no significant difference between PD-NC and PD-SCD. **(C)** A stepwise reduction HC > PD-NC > PD-SCD was observed in ALPS_m. ALPS_L, left DTI-ALPS index; ALPS_R, right DTI-ALPS index; ALPS_m, mean DTI-ALPS index; PD-NC, Parkinson’s disease with normal cognition; PD-SCD, Parkinson’s disease with subjective cognitive decline; HC, healthy controls; ns, not significant. *p < 0.05; ***p < 0.001; ****p < 0.0001.

We validated the ALPS indices using the atlas-based ROI definition method (JHU-ICBM-81) (Supplementary Table 1). The results were highly consistent with the manual ROI method, confirming a significant stepwise decline in the ALPS index across the three groups. To formally evaluate whether the left–right pattern of ALPS indices differed between the two PD groups, a linear mixed-effects model with a group × hemisphere interaction was fitted. The interaction term was statistically significant (*p* = 0.011): in the PD-NC group, ALPS_L was higher than ALPS_R (1.321 ± 0.127 vs. 1.290 ± 0.122; paired *t*-test *p* = 0.004), whereas in the PD-SCD group the two indices did not differ significantly (1.251 ± 0.122 vs. 1.265 ± 0.128; *p* = 0.326) (Supplementary Table 4).

### 3.3 Impact of disease onset side

We further stratified all PD participants by lateralization of initial motor manifestations to test whether the side of motor symptom onset influenced glymphatic function in PD patients. Comparisons between the PD_L and PD_R groups revealed no statistically significant differences in ALPS_L (*p* = 0.0721), ALPS_R (*p* = 0.5606), or ALPS_m (*p* = 0.0907) indices (Supplementary Table 2). These lateralization analyses confirm that the side of symptom onset does not exert a significant effect on the glymphatic system function as measured by the DTI-ALPS index in our cohort.

### 3.4 Diagnostic performance of DTI-ALPS

Given that only the ALPS_L and ALPS_m indices differed significantly between the PD-NC and PD-SCD groups, we evaluated their diagnostic potential using ROC curve analysis (Table 2). The ALPS_L index yielded an AUC of 0.6503 (95% CI: 0.5966–0.7041, *p* < 0.0001), with an optimal cutoff of 1.303 (sensitivity = 0.6711, specificity = 0.5632). Similarly, the ALPS_m index demonstrated modest discriminatory performance (AUC = 0.6452; 95% CI: 0.5907–0.6997, *p* < 0.0001) with an optimal cutoff of 1.254 (sensitivity = 0.5263, specificity = 0.7074).

**TABLE 2:** Receiver operating characteristic (ROC) analyses of DTI-ALPS index in differentiating PD-NC and PD-SCD.

DTI-ALPS	AUC	*P*-value	95% CI	Cutoff value	Sensitivity	Specificity
ALPS_L	0.6503	<0.0001	0.5966–0.7041	1.303	0.6711	0.5632
ALPS_m	0.6452	<0.0001	0.5907–0.6997	1.254	0.5263	0.7074

ROC, Receiver operating characteristic; PD-NC, Parkinson’s disease with normal cognition; PD-SCD, Parkinson’s disease with subjective cognitive decline; AUC, Area under the curve; CI, Confidence interval.

### 3.5 Correlation between DTI-ALPS and cognitive domains

Partial correlation analysis, adjusting for age, sex, education, disease duration, and neuropsychiatric scores, revealed a significant association between the ALPS indices and specific cognitive domains (Figure 4). In PD patients, both the ALPS_L index (*r* = 0.440, *p* < 0.001) and the ALPS_m index (*r* = 0.324, *p* < 0.001) were positively correlated with attention and working memory scores. After FDR correction, these two associations remained significant (ALPS_L: FDR-adjusted *p* < 0.001; ALPS_m: FDR-adjusted *p* < 0.001). These associations remained essentially unchanged when the partial-correlation models were additionally adjusted for LEDD and MDS-UPDRS III scores (ALPS_L: *r* = 0.439; ALPS_m: *r* = 0.328; both *p* < 0.001). No statistically significant correlations were found between ALPS indices and the remaining four cognitive subdomains, including executive function, memory, language, or visuospatial ability.

**FIGURE 4:**
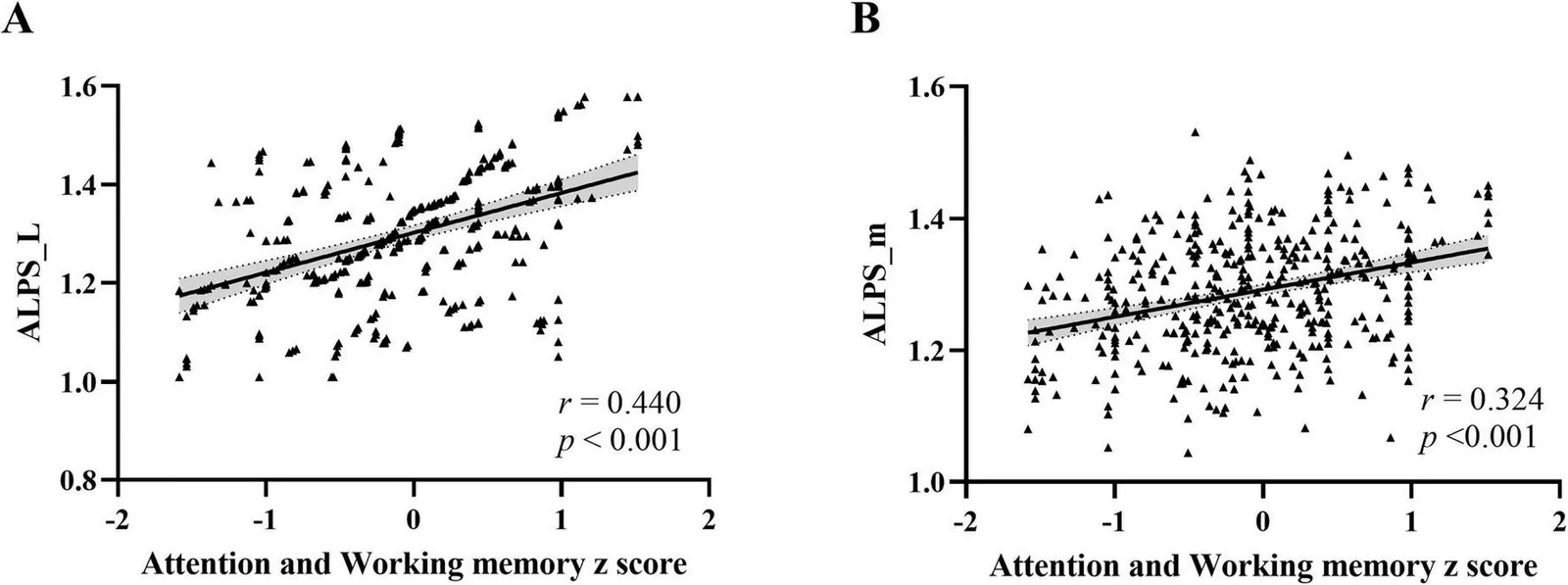
Scatter plots showing partial correlations between DTI-ALPS indices and standardized z-scores of attention and working memory performance across all PD participants. **(A)** Positive partial correlation between ALPS_L indices and attention/working memory z-scores (*r* = 0.440, *p* < 0.001). **(B)** Positive partial correlation between ALPS_m indices and attention/working memory z-scores (*r* = 0.324, *p* < 0.001). ALPS_L, left DTI-ALPS index; ALPS_m, mean DTI-ALPS index.

## 4 Discussion

In present study, we examined diffusion alterations indexed by the DTI-ALPS index across PD patients with and without subjective cognitive decline. As a non-invasive imaging marker, the DTI-ALPS index does not require contrast administration; however, as an indirect measure, it does not directly quantify glymphatic transport. Our intergroup and correlation analyses revealed that lower ALPS indices were associated with PD-SCD and with attention and working-memory performance. These findings suggest that altered directional diffusivity captured by the DTI-ALPS index may be related to early subjective cognitive decline in PD, although the precise biological basis, including any relationship to glymphatic function, remains to be established.

A core finding of our study is the significant reduction in DTI-ALPS indices in PD-SCD patients compared to both PD-NC patients and healthy controls. We observed a stepwise decline in both the left-hemisphere (ALPS_L) and mean (ALPS_m) indices, following the pattern: HC > PD-NC > PD-SCD. This pattern is consistent with altered directional diffusivity in the early stages of PD, particularly in the pre-clinical cognitive impairment phase, and remained robust after adjustment for demographic and clinical covariates. These results align with expanding preclinical and human imaging work implicating compromised glymphatic circulation as a core contributor to neurodegenerative cascades (Taoka et al., 2017; Wu et al., 2025). The glymphatic system functions as a critical clearance pathway for metabolic waste, and its efficiency is essential for maintaining cerebral homeostasis (Nedergaard and Goldman, 2020). PD is characterized by the accumulation and propagation of α-synuclein, and the glymphatic system plays a key role in clearing such metabolic byproducts (Rajai Firouzabadi et al., 2025).

No significant difference was observed in the right-hemisphere ALPS index (ALPS_R) between the PD-NC and PD-SCD groups, contrasting with the significant declines observed in the left-hemisphere and mean indices. A formal interaction test indicated that the left–right pattern differed between the two PD groups (*p* = 0.011). Nevertheless, this asymmetry should be interpreted cautiously: it may reflect differences in measurement variability, local fiber architecture, ROI placement, or statistical power rather than true hemispheric lateralization of perivascular-space function. Our analysis of PD onset side (left vs. right) did not reveal a statistically significant impact on ALPS indices. The potential for laterality in glymphatic function remains a subject of ongoing debate. Some studies suggest asymmetric glymphatic impairment in PD (Li et al., 2025). Another study, aligned with our results, has found no significant association between the side of symptom onset and the ALPS index (Marecek et al., 2025). This discrepancy may arise from the relatively early disease stage of our cohort, variations in sample size, or inherent limitations in the sensitivity of the DTI-ALPS index. It is also plausible that the brain exhibits intrinsic structural and functional asymmetry, as reported in other neurological conditions (Lubben et al., 2021; Zhang et al., 2023; Zhu et al., 2023). Given that α-synuclein propagation may be bilateral from an early stage in PD, and that the glymphatic system functions as an integrated network, the DTI-ALPS index, calculated from relatively large ROIs, might lack the sensitivity to capture micro-regional lateralization effects.

We further evaluated the diagnostic performance of the DTI-ALPS index. Both ALPS_L and ALPS_m showed statistically significant but modest discriminatory performance for PD-SCD (AUC = 0.6503 and 0.6452, respectively). AUC values of approximately 0.65 indicate only modest accuracy at the individual level, and we therefore interpret these findings conservatively: the ALPS index may be best regarded as a potential group-level imaging marker or as one component of a future multimodal risk-stratification model, rather than a stand-alone diagnostic biomarker. The complementary sensitivity/specificity profiles of ALPS_L and ALPS_m may nevertheless be of interest for hypothesis generation in larger, prospective cohorts.

Our partial correlation analysis, after adjusting for potential confounders and FDR correction for multiple comparisons, revealed that DTI-ALPS indices were specifically associated with attention and working memory. This finding is clinically meaningful, as impairment in these domains is often an early hallmark of cognitive decline in PD (Kalbe et al., 2016; Nie et al., 2019). One possible interpretation is that altered water diffusivity captured by the DTI-ALPS index is associated with microstructural changes in prefrontal–striatal circuits, which are essential for attentional control (Sawada et al., 2012). However, given the indirect nature of the DTI-ALPS metric and the cross-sectional design, this remains a speculative hypothesis rather than a demonstrated mechanism. Our results align with previous observations linking DTI-ALPS alterations to attentional deficits in Alzheimer’s disease (Kamagata et al., 2022), but direct evidence of impaired glymphatic clearance underlying these associations is currently lacking.

A significant strength of this study is the robust validation using both manual and atlas-based (JHU-ICBM-81) ROI definition methods. The high consistency between these two approaches underscores the reliability of our findings and minimizes potential bias from ROI placement. While the interpretation of the DTI-ALPS index continues to evolve (Liu et al., 2024; Taoka et al., 2024), our strict adherence to the anatomical assumptions proposed in the original methodology (Taoka et al., 2017), supported by a body of replication studies (Baek et al., 2025; Wu et al., 2025; You et al., 2025), reinforces the validity of our results. Looking ahead, adopting more advanced diffusion models–such as Diffusion Kurtosis Imaging (DKI-ALPS) or skeletonized methods (sk-ALPS)–may further enhance our ability to detect microstructural changes with greater precision (Cao et al., 2024; Csomós et al., 2025; Jensen et al., 2005).

Several limitations of this study should be noted. First, the cross-sectional design precludes definitive causal inferences regarding the longitudinal progression of the observed diffusion alterations. Prospective studies are required to track these changes over time. Second, DTI-ALPS is an indirect, ratio-based diffusion metric obtained at a specific anatomical location; it may be influenced not only by water mobility along perivascular structures but also by the organization and integrity of projection and association fibers, extracellular water content, regional tissue geometry, and other microstructural properties. Recent methodological reviews and a direct comparison with intrathecal contrast-enhanced MRI indicate that the ALPS index should not be equated with a direct measure of glymphatic transport (Mossige et al., 2026; Taoka et al., 2024, 2026). Third, the diffusion acquisition included only one *b* = 0 volume, and susceptibility-induced distortion correction was limited in the present pipeline. Finally, despite controlling for major covariates, we cannot rule out the influence of potential confounding factors such as medication effects or individual genetic variations in AQP4.

In conclusion, this cross-sectional study shows that PD-SCD is associated with a lower DTI-ALPS index, and that this diffusion alteration is related to attention and working-memory performance. The between-group differences remained significant after adjustment for demographic and clinical covariates, and the attention/working-memory association survived correction for multiple comparisons. Because the DTI-ALPS index is an indirect diffusion measure, its precise biological basis and longitudinal significance require further investigation. Future prospective studies are warranted to validate these findings and to clarify the clinical utility of the DTI-ALPS index for cognitive risk stratification in PD.

## Data Availability

The original contributions presented in this study are included in this article/Supplementary material, further inquiries can be directed to the corresponding authors.

## Footnotes

1
https://www.mristudio.org/
