# Supplementary material for: Diffusion tensor image analysis along the perivascular space (DTI-ALPS) in Parkinson’s disease patients with subjective cognitive decline
Source: Front Aging Neurosci. 2026 Sep 18;18:1935631. doi: 10.3389/fnagi.2026.1935631 (PMC13630952; doi:10.3389/fnagi.2026.1935631)
Supplement: Supplementary file 1 [file Table_1.docx]

| Table S1 DTI-ALPS index based on JHU-ICBM-81 | | | | | | | |
| --- | --- | --- | --- | --- | --- | --- | --- |
| DTI-ALPS | PD-NC | PD-SCD | HC | p | p value | | |
|  |  |  |  |  | PD-NC vs PD-SCD | PD-NC vs HC | PD-SCD vs HC |
| ALPS_L | 1.314±0.1399 | 1.269±0.1264 | 1.349±0.1036 | <0.0001 | 0.0014 | 0.0122 | <0.0001 |
| ALPS_R | 1.299±0.1147 | 1.272±0.1169 | 1.339±0.1269 | <0.0001 | 0.0797 | 0.0015 | <0.0001 |
| ALPS_m | 1.306±0.1272 | 1.270±0.1215 | 1.344±0.1151 | <0.0001 | 0.0116 | 0.0044 | <0.0001 |
|  | | | | | | | |
