# Supplementary material for: Diffusion tensor image analysis along the perivascular space (DTI-ALPS) in Parkinson’s disease patients with subjective cognitive decline
Source: Front Aging Neurosci. 2026 Sep 18;18:1935631. doi: 10.3389/fnagi.2026.1935631 (PMC13630952; doi:10.3389/fnagi.2026.1935631)
Supplement: Supplementary file 2 [file Table_2.docx]

| Table S2 DTI-ALPS index between PD_L and PD_R | | | |
| --- | --- | --- | --- |
| DTI-ALPS | PD-L | PD-R | p |
| LPS_L | 1.314±0.1289 | 1.290±0.1265 | 0.0721 |
| ALPS_R | 1.278±0.1189 | 1.271±0.1240 | 0.5606 |
| ALPS_m | 1.296±0.0929 | 1.281±0.0854 | 0.0907 |
