# Supplementary material for: Diffusion tensor image analysis along the perivascular space (DTI-ALPS) in Parkinson’s disease patients with subjective cognitive decline
Source: Front Aging Neurosci. 2026 Sep 18;18:1935631. doi: 10.3389/fnagi.2026.1935631 (PMC13630952; doi:10.3389/fnagi.2026.1935631)
Supplement: Supplementary file 3 [file Table_3.docx]

**Table S3 Adjusted between-group comparisons of DTI-ALPS indices**

| **DTI-ALPS index** | **Unadjusted difference** | **Adjusted β** | **SE** | **95% CI** | **Adjusted p** |
| --- | --- | --- | --- | --- | --- |
| ALPS_L | −0.0700 | −0.0821 | 0.0150 | −0.1116 to −0.0525 | < 0.001 |
| ALPS_R | −0.0245 | −0.0235 | 0.0151 | −0.0532 to 0.0061 | 0.120 |
| ALPS_m | −0.0473 | −0.0528 | 0.0105 | −0.0734 to −0.0322 | < 0.001 |

Abbreviations: PD-NC, Parkinson's disease with normal cognition; PD-SCD, Parkinson's disease with subjective cognitive decline; CI, confidence interval; LEDD, levodopa equivalent daily dose; MDS-UPDRS, Movement Disorder Society Unified Parkinson's Disease Rating Scale.
