# Supplementary material for: Diffusion tensor image analysis along the perivascular space (DTI-ALPS) in Parkinson’s disease patients with subjective cognitive decline
Source: Front Aging Neurosci. 2026 Sep 18;18:1935631. doi: 10.3389/fnagi.2026.1935631 (PMC13630952; doi:10.3389/fnagi.2026.1935631)
Supplement: Supplementary file 4 [file Table_4.docx]

**Table S4 Group-by-hemisphere interaction for DTI-ALPS indices**

| **Group** | **ALPS_L (mean ± SD)** | **ALPS_R (mean ± SD)** | **Paired t-test p** | **Group × hemisphere interaction p** |
| --- | --- | --- | --- | --- |
| PD-NC | 1.3212 ± 0.1266 | 1.2895 ± 0.1220 | 0.0044 | 0.0107 |
| PD-SCD | 1.2512 ± 0.1216 | 1.2650 ± 0.1278 | 0.3255 | — |

Abbreviations: PD-NC, Parkinson's disease with normal cognition; PD-SCD, Parkinson's disease with subjective cognitive decline.
